# A Field Training Guide for Human Subjects Research Ethics

**DOI:** 10.1371/journal.pmed.1000349

**Published:** 2010-10-05

**Authors:** Maria W. Merritt, Alain B. Labrique, Joanne Katz, Mahbubur Rashid, Keith P. West, Joan Pettit

**Affiliations:** 1 Johns Hopkins Berman Institute of Bioethics and Department of International Health, Johns Hopkins Bloomberg School of Public Health, Baltimore, Maryland, United States of America; 2 Department of International Health and Department of Epidemiology, Johns Hopkins Bloomberg School of Public Health, Baltimore, Maryland, United States of America; 3 Department of International Health, Johns Hopkins Bloomberg School of Public Health, Baltimore, Maryland, United States of America; 4 JiVitA Project (USAID-Bangladesh Health and Population Programmes - Johns Hopkins University), Dhaka, Bangladesh; 5 Institutional Review Board Office, Johns Hopkins Bloomberg School of Public Health, Baltimore, Maryland, United States of America

## Abstract

Maria Merritt and colleagues report on a Field Training Guide for Human Subjects Research Ethics that they have developed to help train field workers in ethics for research.

**Summary Points**

- Community trials of interventions to address major global causes of illness and death are often located in low-resource settings, where research findings will be most directly applicable.
- Although investigators delegate research activities involving human subject contact to local field workers, they retain ultimate responsibility for human subject protection and scientific integrity.
- To train every cadre of field worker in research ethics requires simplified training guidelines that can be easily translated and adapted for use in a wide variety of settings and cultural frameworks, especially where field workers have limited formal education.
- Field workers need appropriate training materials, tailored to varying levels of human subject responsibility, that focus on basic principles of community research.
- We have produced a Field Training Guide for Human Subjects Research Ethics, which is freely available to the public. In this article we address how to identify field training needs and meet high standards of research ethics at every level of human subject interaction.

## The Training Gap

Investigators who conduct research with human subjects are responsible for the protection of participants' rights, safety, and welfare, and for scientific integrity [1]–[6]. Each investigator and research site must look to the local laws and ethical standards that apply to their role in a research project [7]. As a matter of routine practice, investigators often delegate to other study team members research activities involving direct contact with human participants. We propose the term *data collector* to designate a distinct role on the study team for field workers who seek informed consent or collect data through direct contact with individuals. When investigators delegate such activities to data collectors, the investigators' ultimate responsibility for human subject protection and scientific integrity is in no way diminished and requires them to train these personnel in the principles and practice of research ethics. Due to a lack of standard training guidelines specific to field workers in low-resource settings, training may vary between sites and principal investigators. Consequently, the effective implementation of research ethics principles may also vary widely and arbitrarily across settings.

**Figure 1:**
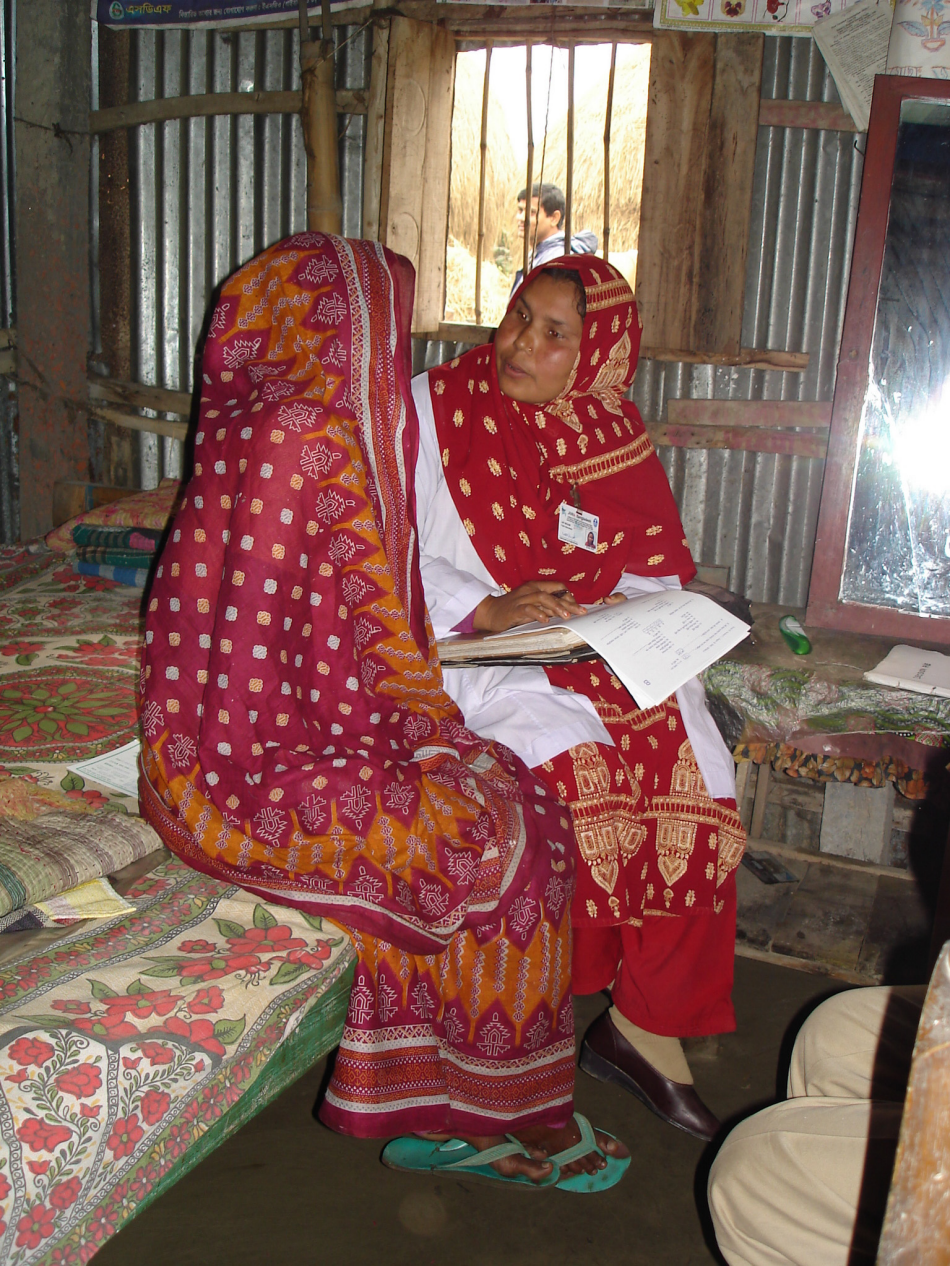
Field interviewer Lipi Begum, of the JiVitA Project in rural Gaibandha, Bangladesh, conducts an enrollment interview with a consented study participant. In addition to building rapport, speaking in the local dialect, using linguistic terms of respect, and clearly explaining the objective of the interview, specific behaviors that convey respect for the participant in the Gaibandha setting are eye contact, the equal position of the interviewer to the participant (seated on the bed), and the prominent display of an identification badge. Specific behaviors should always be tailored to the local setting. For example, while eye contact conveys respect in the setting depicted here, it does not do so universally and would be inappropriate in some other settings. Image Credit: Alain B. Labrique; available under Creative Commons Attribution License.

Several of us (ABL, JK, MR, KPW) (Figure 1) regularly conduct large community trials to evaluate the efficacy and effectiveness of new interventions intended to alleviate major global causes of illness and death, such as diarrheal diseases, respiratory infections, and childhood and maternal undernutrition. We locate our studies at sites where the targeted problem confers a significant burden and research findings will be most directly applicable—most often in low-resource settings, among populations exposed to social or economic stress, and within cultural frameworks that range from sub-Saharan to Southeast Asian [8]–[16]. As our studies can range in size from hundreds to tens of thousands of participants, we find that tailoring research ethics training to the many cadres of workers potentially engaged in human subject contact is both time-consuming and difficult to standardize. We are challenged by the multiplicity of spoken languages, cultures, levels of literacy, and educational backgrounds of the workers hired from the various communities associated with our research sites.

Although training tools exist, some—such as the Collaborative Institutional Training Initiative (CITI) or National Institutes of Health (NIH) online ethics training materials—are too complex for broad application in field settings. Such programs are cumbersome to use in settings with limited access to computers and the internet. Some require institutional affiliations or annual institutional fees. Translations of online training programs into non-English languages are limited, and the attempt to translate them into local languages is challenging and unduly burdensome, potentially resulting in less effective training. Moreover, existing programs are not written at an appropriate educational level for field workers, many of whom have limited formal education. The freely available Family Health International (FHI) Research Ethics Training Curriculum, for instance, is a comprehensive 267-page document designed to train international scientists [17]. The research ethics capacity-building guide offered by the World Health Organization (WHO) is targeted to a similar educational level [18]. By contrast, field workers in low-resource settings need easily accessible, pared-down instruction on the essentials of protecting human subjects, including how to obtain consent in a respectful and diligent manner, how to protect the confidentiality of the data they are collecting, and a few other key ethical concepts. In addition, they need practical support to perform their jobs well, such as guidance on specific behaviors to direct their interaction with human subjects.

## The Field Training Guide

As a first step toward filling the training gap, we used input from a broadly representative group of experienced investigators at our institution to produce a Field Training Guide. In August 2009, we posted the current version of the Guide on our publicly accessible IRB web site (http://www.jhsph.edu/bin/u/p/Field%20Guide_25Feb10.pdf) [19]. Investigators working in various countries and situations were encouraged to adapt the delivery of content to their specific projects.

The Guide is innovative in two primary ways. First, it identifies the distinct role of *data collector* and situates the responsibilities of the role within the institutional context governing the activities of the entire research team [20]. The Guide defines *data collectors* as study team members “who will (1) obtain informed consent from research participants, or (2) collect data from human subjects through individual or focus group interviews, testing, physical measurements, or other procedures involving direct contact” [19]. As an agent representing study investigators and the institutions charged with ethical oversight of the research protocol, the *data collector* bears two weighty responsibilities: to act respectfully and otherwise appropriately toward each study participant with whom he or she has contact for research purposes; and to safeguard the confidentiality and integrity of the data that he or she collects [19].

Second, the Guide presents the two main responsibilities of the data collector's role in concrete terms that trainers can readily convey to workers in the field. This includes detailed instruction on specific behaviors that promote basic ethical principles: for example, paying attention to body language when seeking informed consent or recording interview responses, and safeguarding data collection sheets to protect the confidentiality of personal information [19]. The Guide conveys the importance of correct, accurate data recording and careful, systematic transmittal of data records. It also addresses the cultural challenge of encouraging data collectors to admit to mistakes or inadvertent procedural lapses, or to ask questions about things they do not understand.

Our IRB Office now routinely refers investigators to the Guide as an approved curriculum in human subjects research training for study staff. Users have translated the Guide into French, Mandarin, and Thai (Text S2–S4). Bangla and Nepali translations are in progress. The Nepali translation will be used to test and obtain feedback on the Guide's content while training workers for new trials in Nepal.

## Additional Training Needs

Our ongoing experiences in the field and in the IRB review process indicate needs for additional materials to support investigators' delivery of ethical training to field workers.

### Levels of Authority in Interaction with Human Subjects

The consenting process should always be carried out by a responsible cadre of workers articulate in the local language and culture. In some research settings, workers authorized to administer informed consent are the same individuals who collect all the data, whereas in other settings consent is only within the purview of supervisor-level staff. The current version of our Guide is oriented toward this higher-level cadre of workers. In settings where data are collected by a distinct cadre of workers, usually less educated, who are not authorized to administer consent, a need exists for field training materials targeted specifically to them. Investigators could use a checklist to assess the level of human subject interaction a particular cadre of staff will be engaged in, and thus tailor the level of training needed for that cadre above a threshold of basic respect for persons and confidentiality. For example, interviewers collecting in-depth data on socioeconomic characteristics or sexual behaviors have a different level of responsibility than workers doing simple head counts, and need to understand their ethical obligations in greater depth.

### Orientation to Basic Principles of Community Research

Technologically and culturally appropriate training materials are needed to orient higher-level cadres of field workers to basic principles of community research, such as equipoise (to explain that research would not be ethical if an answer were known to exist), randomization, and placebo controls (how it is that some people may get an active substance and others not). Training should prepare field workers to respond appropriately when participants perceive them as medical workers and ask them for advice exceeding their level of competence, or when they feel compelled to intervene in a situation of acute medical need but are neither trained nor mandated to do so. Investigators need to define lines of recourse for field workers who find themselves facing such dilemmas [21],[22].

### Social Complexities of Community Research

Approaching subjects to seek informed consent or collect data in community research usually involves contact not only with the prospective subject but also with senior decision makers in the family. The very event of a home visit by a field worker has the potential to breach a subject's confidentiality by exposing private circumstances to the neighborhood, as when a field worker tests a subject for pregnancy and brings antenatal supplements on subsequent visits. Field workers in community settings are often pressed by local government officials, opinion leaders, family heads, or NGO workers to divulge information about study subjects, as in studies of sexually transmitted conditions or other sensitive matters. Field workers need to be trained to manage these complex social situations so as to protect subjects' privacy and the confidentiality of personal information.

## Opening a Conversation on Field Training Tools for Research Ethics

Our Field Training Guide is a first step toward developing locally adaptable research ethics training tools for study teams working in a variety of settings around the world. To the best of our knowledge, following an extensive search, no other comparably simple field training guide is publicly available. We intend our Guide to be suitable for adaptation by investigators beyond our institution, assuming that they already have in place an organizational infrastructure through which they normally train and supervise field personnel in the activities necessary to conduct research. We encourage readers to examine the Guide, try it out, translate it, and identify potential improvements. A welcome step forward would be systematic evaluation of the Guide, or of users' adaptations thereof, as compared with other training tools. We invite other investigators and institutions to join us in conversation about how to address field training needs so as to meet high standards of research ethics at every level of human subject interaction.

## Supporting Information

Text S1 JHSPH Field Training Guide: English.
(0.04 MB PDF)

Text S2 French translation of JHSPH Field Training Guide courtesy of TransPerfect Translations.
(0.05 MB PDF)

Text S3 Mandarin translation of JHSPH Field Training Guide courtesy of Youfa Wang.
(0.31 MB PDF)

Text S4 Thai translation of JHSPH Field Training Guide courtesy of David Celentano and Louise Walshe.
(0.17 MB PDF)

## Abbreviations

CITI: Collaborative Institutional Training Initiative
FHI: Family Health International
JHSPH: Johns Hopkins School of Public Health
NIH: National Institutes of Health
WHO: World Health Organization
