# Supplementary material for: A Field Training Guide for Human Subjects Research Ethics
Source: PLoS Med. 2010 Oct 5;7(10):e1000349. doi: 10.1371/journal.pmed.1000349 (PMC2950122; doi:10.1371/journal.pmed.1000349)
Supplement: Text S1 — JHSPH Field Training Guide: English. (0.04 MB PDF) [file pmed.1000349.s001.pdf]

JHSPH HUMAN SUBJECTS RESEARCH ETHICS
FIELD TRAINING GUIDE

This guide is intended to be used as a tool for training individuals who will be "engaged" in some aspect of a human subject research interaction or intervention. It is directed, in particular, to Johns Hopkins principal investigators who are responsible for training of study team members who will (1) obtain informed consent from research participants, or (2) collect data from human participants through individual or focus group interviews, testing, physical measurements, or other procedures involving direct contact, hereafter called a "data collector". The content and language level of this guide is specifically worded to help the investigator convey basic research principles and behavior that accords with those principles to data collectors. We encourage users who translate the document into local languages to submit those translations (with certifications of the translator's qualifications) to our office (irboffice@jhsph.edu) so we can make them available to other investigators.

TABLE OF CONTENTS

1. Ethical Interaction with Human Participants
    a. Role of the Data Collector
    b. Importance of Respect
    c. Voluntary Participation
    d. Informed Consent
    e. Vulnerable Populations
    f. Personal Privacy
    g. Protection of Personal Information
    h. Response to Participant Questions

2. Data Integrity
    a. Respect for the Science of the Study
    b. Collecting, Recording, and Storing Study Data
    c. Deviations from Study Procedures

Copyright 2010 by Johns Hopkins University. All Rights Reserved.

1. Ethical Interaction with Human Participants

a. Role of the Data Collector

The person who collects information on behalf of a research team is an "ambassador" for the study. The data collector is sometimes the only person on the study team with whom a research participant will come into contact. People who come into contact with that person will have a good impression of the study or a bad impression of the study, depending on how the data collector presents him- or herself.

The data collector has the responsibility for making sure that the information collected for the study comes from individuals who understand what they are agreeing to do. In addition, the data collector must ensure that the information collected and recorded is accurate and protected from loss. Otherwise, the study objectives will not be achieved. To be successful, the data collector must carefully follow the research plan, study operations manual and other procedures involving contact with human participants.

b. Respect

Each person who is part of the research team must show respect for:

- the goals of the research project,
- the leaders of the project,
- the individual study participant,
- the participant community and
- the data collected that will help achieve project objectives.

The scientific project has the potential to benefit the community that will be studied, but only if the research team is able to complete all the parts of the study.

Each individual research team member must conduct all interactions with members of the participant community with respect. This includes respecting the participant community's culture, gender, age, social status, religion and other characteristics that make people different from each other, as well as their right to ask questions. The individual does not have to help the research team by participating in the study, and they will not do so if the research team is not respectful of the participant's right to say "yes" or "no", or to receive honest and true information from all team members

The data collector conveys to each participant the importance of the study purpose by collecting data in a professional and respectful manner. For example:

- Always be polite to the participant whether or not she or he agrees to participate in the study.

Copyright 2010 by Johns Hopkins University. All Rights Reserved.

- If the data collector is asking questions, ask them in a clear voice.
- Record the information neatly on the data collection sheets.
- If the participant asks a question, provide an answer that is correct. If you do not know the answer, and it is possible for you to obtain the information from your supervisor, tell the participant that you will obtain an answer to their question and that you will let them know what you find out.
- Thank the participants once they have completed the study procedures.

c. Voluntary Participation

No individual person is required to participate in a research project. If the study includes an informed consent process, then each person approached by a study team member has the right to refuse to hear about the study, and the right to refuse to join the study. Even if a person joins the study, he or she may refuse to answer specific questions in a survey or questionnaire, refuse to give a specimen or refuse to take a test, and may decide to withdraw from a study at any time.

The research team member who obtains informed consent from participants is responsible for ensuring that the individual understands what the study is about and truly agrees to join the study and is not joining because they are afraid not to, or feel forced to join.

d. Informed Consent

Providing correct, factual information to persons being approached to join a study is an essential part of human subjects research. Informed consent is an ongoing process that begins with the research team member explaining the study to the participant. Informed consent does not end with the participant signing the consent form and agreeing to be in the study. The process of informed consent continues throughout the study; for example, it can occur each time a data collector and participant interact. There is no true “consent” to join a study if a person does not adequately understand what is being asked of them. The investigative team determines ahead of time what is “adequate” and how that information should be conveyed. The job of the person conducting informed consent is to present information about the study to the potential participant in language that s/he can understand, and in a way that reveals the study purpose, procedures, potential risks and benefits of the study. The language and style of communication should enable a participant to understand. That discussion should give the participant enough time to ask questions and to think about the decision whether or not to join the study.

Sometimes it is important to check in with the participant from time to time to make sure that the participant continues to understand what the study is about, or what it involves. The study team member who is obtaining consent may have to ask the participant questions to see what the participant has learned, and whether the participant has the correct understanding. The research team

Copyright 2010 by Johns Hopkins University. All Rights Reserved.

member should also be aware of the participant's body language, as the participant may look physically uncomfortable or confused, but not say so. If these responses are observed, the study team member should notify his/her supervisor for further guidance.

e. Vulnerable Populations

Some people need extra attention and care when approached to participate in a research project because they have conditions that make it difficult for them to understand what is being told to them and to provide informed consent. For example, children need extra "protection" and it is important that their parents make certain decisions for them. Adults who have dementia may not understand what you are asking them to do. The research plan and the operations manual, developed by the investigators, should tell you how to approach these individuals, and if they are to be included in the study. Research team members must be very careful to follow the rules when enrolling vulnerable populations because most of the time they cannot make decisions for themselves. If the study will include people who cannot make decisions for themselves, an authorized caregiver or other proper representative must be available to decide for them.

f. Personal Privacy

Individuals have a right to privacy that the research team must understand and respect. Even if the culture does not promote or generally give recognition to the concept of "privacy", it is important that the right to privacy be protected to every possible extent. For example, it may be the custom that no one enters another person's home without being invited. The study team must honor this custom. Or, the visit of a data collector to a home may attract curious onlookers, whose presence may be undesirable and will distract the data collector and participant from focusing on the important process of data collection. The study team must anticipate this problem and minimize it. When a data collector requests privacy, that action assures the respondent that the data collector respects that the home is where the family lives; it is not a public space.

Research team members must also respect the participant's personal privacy by not causing them any unnecessary personal embarrassment or discomfort. Interviews involving sensitive information should take place where other people cannot hear the questions or responses. Physical examinations should not occur where other people can watch. Also, there are certain things that are considered to be "private", such as sexual activity, personal health, or thoughts that one might not want to talk about in public.

Copyright 2010 by Johns Hopkins University. All Rights Reserved.

g. Protection of Personal Information

When a study participant discloses personal information about him or herself to a data collector, that participant is at risk of having highly confidential information become "public". That is, s/he risks losing confidentiality. The risk is that if someone outside the study learns about the private information, bad things could happen to the participant, like embarrassment, loss of employment, legal problems, or social damage. The research team is responsible for protecting the participant from this kind of injury.

After a study participant has provided information, that personal information must be kept safe. No one without the proper authority should see or have access to the information. If the information is written on paper, then that paper should be protected until it is locked up in a cabinet. It must only be seen and processed by study staff who are authorized by the study investigators to handle the information. If the information is electronic, then all necessary precautions should be taken to make sure that no unauthorized person can access it.

Sometimes a random study number is used to identify the data so that no one will know which participant the data came from. Any document that links a number with the name of the person it is assigned to must be locked up and kept safe and secure. The research plan and operations manual must be followed to make sure that the study data are protected exactly as prescribed in the manuals.

h. Response to Participant's Questions

A data collector will meet many people, including prospective participants, existing study participants and curious onlookers not involved in the study, who will have questions about the study. Some people will not understand what "research" is, or will not know anything about the researchers who are leading this project. They may have all sorts of questions, some of which may not have anything to do with the study procedures at all.

Investigators will train data collectors to address the many concerns that are likely to be expressed by people. This is because, in the "field" on a day to day basis, it is the data collector who represents the study when talking with possible participants and the community at large. It is important that the data collector show proper respect to all individuals and do one's best to address concerns. A data collector must be patient and answer any question that a participant asks, so long as s/he knows the answer! A data collector should never answer questions for which the answers are not clearly known, because giving wrong information can be worse than giving no information, at least temporarily. If you are a data collector and a participant asks a question and you are not sure of the answer, here's what should happen: you should tell the person that you do not have a confident answer to the question; that you will ask the study supervisor

Copyright 2010 by Johns Hopkins University. All Rights Reserved.

the question; and that you will pass that answer on to the participant. This is very important because it shows respect to the participant and it makes sure that the information you pass on to the participant is accurate. When you think the participant has no more questions, you may ask, “Do you have any other questions?” to make sure that all questions have been addressed. If there are no more questions, then you may proceed.

2. Data Integrity

a. Respect for the Science of the Study

Data are the “product” of research. It is very important that the information collected, recorded, and stored by the data collectors is correct. Scientists will use these data to answer the research questions identified in the research plan. If the data are wrong, then the answers that the scientists produce will also be wrong. People whose lives may be affected by the results of the study may be put at risk, because the answers and actions that follow will be wrong. So it is very important that all data at all times are collected properly, recorded properly, and stored properly. If you make a mistake doing any of these things, it is important that you tell your supervisor right away so that the investigators or research team leaders know about it. They may be able to fix the problem, or the will know that some data may not be usable.

b. Collecting, Recording, and Storing Study Data

The research plan spells out the project objectives and how the research team will reach those objectives. The details of data collection and recording study data are included, and usually the study operations manual goes into more detail about how those jobs will be done. The data collector must understand exactly how the data should be collected, and how they are recorded. The research team leaders will train the data collectors on this process. If the data collectors have any questions, they must not be afraid to ask them. In truth, if the data collectors do not ask questions when they are unsure of how things should be done, they will not be able to make sure that the data are correct.

Once the training is complete, the data collectors begin their job. Good data collection means following the instructions and accurately completing the data collection sheet. Proper recording includes making sure that the answers to questions are written in a legible and clear way. The data collector must record the information with honesty and accuracy. Extra information that is not identified in the data collection sheet should not be included. For example, if there is no space for “name” or “address”, then these data should not be recorded on the data collection sheet. No information should be “made up” and recorded on the data collection sheet.

Copyright 2010 by Johns Hopkins University. All Rights Reserved.

Proper storing of data means that all safety precautions should be taken while transporting the data to the ultimate storage place. Data collectors should not put the collection sheets down where they might be lost, stolen, or read by someone outside the research team. The data collection sheets should be given to the person responsible for storage, and that person should follow all the instructions to protect data confidentiality. If data are collected electronically, the same principles and rules of honesty, protection and care must be followed.

c. Deviations from Study Procedures

Sometimes a data collector is not able to follow study procedures through no fault of his or her own, and sometimes a data collector may make a mistake. It is very important to let the research leaders know about these problems because the research leaders have the responsibility to report these kinds of problems to the reviewing Institutional Review Board (IRB). There is no shame in reporting these kinds of problems. They happen all the time. It is not good, however, if the data collector fails to report these problems because it could mean that the data are not good, or that a participant has a problem with the study. It also means that the study supervisor will not be able to complete the report to the IRB.

A good data collector will communicate these issues to his or her supervisor and let that person decide what action to take.

Copyright 2010 by Johns Hopkins University. All Rights Reserved.
