# Supplementary material for: A Field Training Guide for Human Subjects Research Ethics
Source: PLoS Med. 2010 Oct 5;7(10):e1000349. doi: 10.1371/journal.pmed.1000349 (PMC2950122; doi:10.1371/journal.pmed.1000349)
Supplement: Text S2 — French translation of JHSPH Field Training Guide courtesy of TransPerfect Translations. (0.05 MB PDF) [file pmed.1000349.s002.pdf]

ÉTHIQUE PRÔNÉE PAR L'ÉCOLE DE SANTÉ PUBLIQUE JOHNS-HOPKINS (JHSPH) EN CE QUI CONCERNE LA RECHERCHE SUR LES SUJETS HUMAINS

MANUEL DE FORMATION TERRAIN

Ce manuel doit être utilisé comme un outil de formation du personnel engagé dans un aspect quelconque de l'interaction avec la recherche sur des sujets humains, ou de l'intervention dans ce cadre. Il s'adresse en particulier aux investigateurs principaux de Johns-Hopkins, ci-après dénommés « collecteurs de données », chargés de la formation des membres de l'équipe d'étude, qui (1) obtiendront le consentement éclairé des participants à l'étude ou (2) recueilleront des données auprès des participants humains à partir d'entretiens individuels ou de groupes de discussion, de tests, d'examens physiques ou d'autres procédures impliquant un contact direct. Les informations et le niveau de langue du présent manuel sont spécialement formulés de manière à aider l'investigateur à transmettre aux collecteurs de données les principes de recherche de base ainsi que le comportement à adopter au vu de ces principes. Nous encourageons les utilisateurs qui traduisent le présent document dans la langue de leur pays à envoyer ces traductions (accompagnées de justificatifs des qualifications du traducteur) à notre bureau (irboffice@jhsph.edu) de manière à ce que nous puissions les mettre à disposition des autres investigateurs.

SOMMAIRE

1. Interaction éthique avec les participants humains
    - a. Rôle du collecteur de données
    - b. Importance du respect
    - c. Participation volontaire
    - d. Consentement éclairé
    - e. Populations vulnérables
    - f. Confidentialité
    - g. Protection des données personnelles
    - h. Réponses aux questions des participants

2. Intégrité des données
    - a. Respect des procédures de recueil des données
    - b. Recueil, consignation et archivage/enregistrement des données d'étude
    - c. Non observance des procédures d'étude

1. Interaction éthique avec les participants humains

a. Rôle du collecteur de données

La personne qui rassemble les informations pour l'équipe de recherche est un « ambassadeur » de l'étude. Le collecteur de données est parfois la seule personne de l'équipe d'étude avec qui le participant à la recherche entrera en contact.
Ceux qui entrent en contact avec cette personne auront une bonne ou une mauvaise impression de l'étude, selon la manière dont le collecteur de données se présentera.

Le collecteur de données est responsable de s'assurer que les informations rassemblées pour l'étude sont fournies par des interlocuteurs qui comprennent à quoi ils s'engagent. En outre, le collecteur de données doit vérifier que les informations recueillies et consignées sont exactes et protégées contre toute perte. Autrement, les objectifs de l'étude ne seront pas atteints. Pour atteindre son but, le collecteur de données doit suivre méthodiquement le plan d'étude, le guide opérationnel d'étude et les autres procédures impliquant un contact avec des participants humains.

b. Respect

Chaque membre de l'équipe de recherche doit se montrer respectueux :

- des objectifs du projet de recherche ;
- des chefs de projet ;
- de chaque participant à l'étude ;
- du groupe des participants ;
- des données rassemblées qui aideront à atteindre les objectifs du projet.

Le projet scientifique peut potentiellement bénéficier au groupe étudié, mais seulement si l'équipe de recherche est capable de valider toutes les étapes de l'étude.

Chaque membre de l'équipe de recherche doit interagir respectueusement avec les membres du groupe de participants. Cela induit de respecter la culture, le sexe, l'âge, le statut social, la religion et les autres caractéristiques du groupe de participants qui rendent chaque personne unique, ainsi que de respecter leur droit de poser des questions. Personne n'est obligé d'aider l'équipe de recherche en participant à l'étude et personne ne le fera si l'équipe n'est pas respectueuse du droit du participant de dire « oui » ou « non », ou de recevoir des informations honnêtes et exactes de la part de tous les membres de l'équipe.

Le collecteur de données véhicule l'importance de la finalité de l'étude auprès de chaque participant en recueillant ses données avec professionnalisme et respect. Par exemple :

- Soyez toujours poli envers le participant, qu'il accepte ou non de participer à l'étude.

- Si le collecteur de données pose des questions, il doit s’exprimer de manière intelligible.
- Consignez proprement les informations sur les fiches de recueil des données.
- Si le participant pose une question, fournissez-lui une réponse juste. Si vous ne connaissez pas la réponse et que votre responsable peut vous renseigner, dites au participant que vous allez chercher la réponse et lui en faire part ultérieurement.
- Remerciez les participants une fois les procédures d’étude validées.

c. Participation volontaire

Personne n’est obligé de participer à un projet de recherche. Si l’étude comprend un processus de consentement éclairé, tout interlocuteur sollicité par un membre de l’équipe d’étude a le droit de refuser d’être informé sur l’étude et de ne pas y participer. Même en cas de participation, la personne peut refuser de répondre à des questions précises d’une enquête ou d’un questionnaire, de fournir un échantillon ou de se soumettre à un test et décider à tout moment de se retirer de l’étude.

Le membre de l’équipe de recherche qui obtient le consentement éclairé des participants est chargé de s’assurer que chacun comprend le sujet de l’étude et accepte sans équivoque d’y participer, et ce, non pas parce qu’il redoute de ne pas le faire ou qu’il s’y sent contraint et forcé.

d. Consentement éclairé

Dans le cadre de la recherche sur des sujets humains, il est essentiel de fournir des informations correctes et factuelles à ceux dont le concours est sollicité pour participer à l’étude. Le consentement éclairé est un processus continu qui commence dès l’explication de l’étude au participant par le membre de l’équipe de recherche. Ce consentement ne s’achève pas une fois que le participant a signé le formulaire de consentement et accepté de rentrer dans l’étude. Le processus de consentement éclairé se poursuit tout au long de l’étude ; par exemple, il peut être mis en œuvre à chaque fois qu’un collecteur de données et un participant interagissent. Il n’existe pas de « consentement » réel pour participer à une étude si l’interlocuteur sollicité ne comprend pas bien ce qui lui est demandé. L’équipe de recherche détermine à l’avance ce qu’est une « bonne » compréhension et comment les informations doivent être transmises. Le membre de l’équipe qui mène le processus de consentement éclairé est chargé de donner des informations sur l’étude au participant potentiel dans une langue qu’il comprend et de manière à mettre en évidence la finalité, les procédures ainsi que les risques et les bénéfices éventuels de l’étude. La langue et le style de communication doivent faciliter la compréhension d’un participant. Cette discussion doit laisser assez de temps au participant pour poser des questions et réfléchir à sa décision concernant sa participation à l’étude.

Parfois, il est important de s’assurer régulièrement auprès du participant qu’il comprend toujours le sujet ou les implications de l’étude. Il se peut que le membre de l’équipe de recherche qui obtient le consentement ait à poser des questions pour voir ce qu’a appris le participant et s’il a bien compris. Le membre de l’équipe de recherche doit également savoir décrypter le langage corporel du participant, car ce dernier peut sembler physiquement mal à l’aise ou confus sans le dire. Si le membre de l’équipe d’étude constate ce comportement, il doit demander la marche à suivre à son responsable.

e. Populations vulnérables

Certaines personnes doivent être sollicitées avec une attention et une prévenance toutes particulières pour participer à un projet de recherche parce qu'ils ne sont en mesure ni de comprendre aisément ce qui leur est dit, ni de donner un consentement éclairé. Par exemple, les enfants ont besoin d'une « protection » supplémentaire et il est important que leurs parents prennent certaines décisions pour eux. Les adultes atteints de démence ne comprennent pas forcément ce qui leur est demandé. Le plan d'étude et le guide opérationnel développés par les investigateurs doivent vous indiquer comment aborder ces personnes et si elles doivent être incluses dans l'étude. Les membres de l'équipe de recherche doivent suivre les règles à la lettre dans le cadre de l'inclusion de personnes vulnérables car la plupart du temps, elles ne sont pas capables de prendre de décisions concernant leur propre personne. Si l'étude doit inclure des sujets incapables de prendre leurs propres décisions, des aidants habilités ou autres représentants compétents doivent pouvoir décider à leur place.

f. Confidentialité

Chacun dispose d'un droit de confidentialité que l'équipe de recherche doit comprendre et respecter. Même si la culture n'encourage pas ou, de manière générale, ne reconnaît pas le concept de « confidentialité », le droit de confidentialité doit être protégé autant que possible. Par exemple, il est d'usage que personne ne pénètre chez quelqu'un d'autre sans y avoir été invité. L'équipe de recherche doit respecter cet usage. Dans ce cas précis, la visite d'un collecteur de données dans un foyer peut attirer des badauds dont la présence peut être indésirable et détournera l'attention du collecteur de données et du participant concentrée sur l'important processus de recueil des données. L'équipe de recherche doit anticiper ce problème et le minimiser. Lorsqu'un collecteur de données demande la confidentialité, cela garantit au répondant qu'il respecte le fait que le foyer est le domicile de la famille ; il ne s'agit pas d'un espace public.

Les membres de l'équipe de recherche doivent également respecter la confidentialité du participant en ne lui causant pas de gêne ou d'embarras superflus. Les entretiens impliquant des informations sensibles doivent se tenir là où personne d'autre ne peut entendre les questions ou les réponses. Les examens physiques ne doivent pas être réalisés à la vue de tous. De plus, certaines choses telles que l'activité sexuelle, la santé ou les avis personnels sont considérées « privées » et chacun est susceptible de ne pas vouloir en parler en public.

g. Protection des données personnelles

Lorsqu'un participant à l'étude divulgue des informations privées le concernant à un collecteur de données, il court le risque que ses informations de la plus haute confidentialité soient rendues « publiques ». C'est-à-dire qu'il risque de perdre sa confidentialité. Le risque est qu'une personne extérieure à l'étude découvre ses informations privées et nuise au participant en lui causant des ennuis, des problèmes juridiques ou du tort sur le plan social, ou encore en lui faisant perdre son emploi. L'équipe de recherche a pour responsabilité de protéger le participant de ce genre de préjudice.

Une fois que le participant à l'étude a fourni des informations, elles doivent être tenues confidentielles. Seules les personnes dûment habilitées doivent consulter ou avoir accès à ces informations. Si les informations sont consignées sur papier, les feuillets doivent être protégés jusqu'à ce qu'ils soient mis sous clé dans une armoire. Les informations ne doivent être consultées et traitées que par le personnel d'étude habilité à utiliser ces informations par les investigateurs de l'étude. Si les informations sont sous forme électronique, toutes les précautions nécessaires doivent être prises pour garantir que seules les personnes habilitées peuvent y accéder.

Parfois, un numéro d'étude aléatoire sert à identifier les données de manière à ce que personne ne puisse connaître l'identité du participant qui a fourni ces données. Tout document qui associe un numéro au nom de la personne à laquelle il est attribué doit être mis sous clé et placé en lieu sûr. Il faut suivre le plan de recherche et le guide opérationnel pour s'assurer que les données d'étude sont protégées exactement tel que cela est formulé dans les guides.

h. Réponses aux questions des participants

Un collecteur de données rencontre beaucoup de gens, y compris des participants potentiels, les participants à l'étude existants et les spectateurs curieux qui ne sont pas concernés par l'étude et ont des questions à son sujet. Certains interlocuteurs ne comprennent pas ce que recouvre le terme « recherche », ou bien ne savent rien des chercheurs qui mènent ce projet. Ils peuvent poser toutes sortes de questions dont certaines peuvent être sans aucun rapport avec les procédures de l'étude.

Les investigateurs forment les collecteurs de données à répondre aux nombreuses questions susceptibles d'êtres posées. La raison en est que sur le « terrain », au quotidien, c'est le collecteur de données qui représente l'étude à travers le discours qu'il tient à d'éventuels participants et à toute la communauté. Il est important que le collecteur de données témoigne du respect dû à ses interlocuteurs et fasse de son mieux pour répondre à leurs préoccupations. Un collecteur de données doit être patient et répondre à toute question d'un participant, dans la mesure où il connaît la réponse bien sûr ! Un collecteur de données ne doit jamais répondre à des questions lorsqu'il n'est pas certain des réponses, car fournir des informations erronées peut être pire que de ne pas en fournir du tout, au moins provisoirement. Si vous êtes collecteur de données, qu'un participant vous pose une question et que vous n'êtes pas sûr de la réponse, voilà la marche à suivre : vous devez lui dire que vous n'avez pas de réponse certaine à cette question, que vous allez demander à votre responsable puis que vous lui ferez part de la réponse ultérieurement. C'est très important car cela démontre le respect dont vous faites preuve envers le participant et ceci garantit l'exactitude des informations qui lui sont transmises. Si vous pensez que le participant n'a plus de questions, vous pouvez demander : « Avez-vous d'autres questions ? » pour vous assurer que toutes les questions ont été abordées. En l'absence d'autres questions, vous pouvez continuer.

2. Intégrité des données

a. Respect des procédures de recueil des données

Les données sont les « produits » de l'étude. Il est essentiel que les informations recueillies, consignées et archivées/enregistrées par les collecteurs de données soient correctes. Les scientifiques exploiteront ces données pour répondre aux questions de recherche identifiées dans le plan de recherche. Si les données sont erronées, les réponses que les scientifiques produiront seront également fausses. Cela peut exposer à un risque ceux dont la vie peut être affectée par les résultats de l'étude, dans la mesure où les réponses et les actions qui en découlent seront erronées. Il est donc impératif que toutes les données soient toujours recueillies, consignées et archivées/enregistrées correctement. Si vous vous trompez lors de l'une de ces étapes, il est important que vous le disiez immédiatement à votre responsable de manière à ce que les investigateurs ou les chefs de l'équipe de recherche soient informés. Ils peuvent peut-être régler le problème ou ils sauront que certaines données ne sont pas exploitables.

b. Recueil, consignation et archivage/enregistrement des données d'étude

Le plan de recherche énonce les objectifs du projet et la méthode appliquée par l'équipe de recherche pour les atteindre. Le recueil et la consignation des données d'étude y sont présentés et, en général, le guide opérationnel d'étude explique plus en détail comment procéder. Le collecteur de données doit comprendre le mode de recueil et de consignation des données attendu. Les chefs de l'équipe de recherche forment les collecteurs de données à ce processus. Si les collecteurs de données ont la moindre question, ils ne doivent pas hésiter à la poser. En vérité, si les collecteurs de données ne posent pas de questions quand ils ne sont pas sûrs de la manière de procéder, ils ne peuvent pas s'assurer de l'exactitude des données.

Une fois la formation terminée, les collecteurs de données commencent leur travail. Les données sont convenablement recueillies quand les collecteurs suivent les instructions et remplissent la fiche de recueil des données avec précision. Pour bien consigner les données, il faut s'assurer que les réponses sont rédigées de manière lisible et claire. Le collecteur de données doit consigner les informations avec honnêteté et exactitude. Les informations complémentaires qui ne sont pas identifiées sur la fiche de recueil des données ne doivent pas être incluses. Par exemple, s'il n'y a pas d'espace pour « nom » ou « adresse », ces renseignements ne doivent pas être consignés sur la fiche de recueil des données. Aucune information ne doit être « fabriquée » et consignée sur la fiche de recueil des données.

Pour archiver/enregistrer convenablement les données, il faut prendre toutes les précautions nécessaires en matière de sécurité lors de leur transport vers le lieu d’archivage/d’enregistrement final. Les collecteurs de données ne doivent pas poser les fiches de recueil là où elles pourraient être perdues, volées ou lues par quelqu’un d’étranger à l’équipe de recherche. Il faut remettre les fiches de recueil des données au responsable de l’archivage/de l’enregistrement qui doit suivre toutes les instructions visant à protéger la confidentialité des données. Si les données sont recueillies par informatique, les mêmes principes et règles d’honnêteté, de protection et d’attention doivent être appliqués.

c. Non observance des procédures d’étude

Parfois, un collecteur de données pour des raisons indépendantes de sa volonté n’est pas en mesure de suivre les procédures d’étude, et il peut également se tromper. Il est essentiel de signaler ces problèmes aux chefs de l’équipe de recherche parce qu’ils sont chargés de communiquer ce type de problèmes au comité d’éthique institutionnel (CEI). Il n’y a aucune honte à signaler ce type de problèmes. Ils sont monnaie courante. Par contre, le collecteur de données commet une erreur en gardant ces problèmes pour lui parce que cela pourrait signifier que les données sont erronées ou qu’un participant connait un problème avec l’étude. Cela signifie également que le responsable d’étude ne sera pas en mesure de compléter le rapport à soumettre au CEI.

Un bon collecteur de données communique ces problèmes à son responsable et le laisse décider des mesures à prendre.
