# Supplementary material for: A Field Training Guide for Human Subjects Research Ethics
Source: PLoS Med. 2010 Oct 5;7(10):e1000349. doi: 10.1371/journal.pmed.1000349 (PMC2950122; doi:10.1371/journal.pmed.1000349)
Supplement: Text S3 — Mandarin translation of JHSPH Field Training Guide courtesy of Youfa Wang. (0.31 MB PDF) [file pmed.1000349.s003.pdf]

美国约翰霍普金斯大学公共卫生学院
与人类相关研究的伦理学培训指南
(9/2009)

本指南是用于培训将要参加与人类有关研课题的工作者。本指南主要是帮助约翰霍普金斯大学的项目负责人对其他相关研究人员进行培训，使他们的研究行为符合伦理学要求，并以符合伦理要求的方式去：1) 取得研究对象的知情同意， 2) 通过对研究对象进行个人或小组的面谈、检查、身体测量，或以其他直接接触的渠道收集数据 (以下简称“调查员”)。本指南所采用的特定的内容和语言水平，是为了帮助项目负责人向调查员传达基本的研究准则和相应的行为标准。

目 录

1. 在和研究对象接触中的道德准则

a. 调查员的任务
b. 尊重待人的重要性
c. 自愿参与
d. 知情同意
e. 弱势人群
f. 个人隐私
g. 个人信息的保护
h. 回应研究参与者 (研究对象) 的问题

2. 数据的完整性

a. 尊重研究的科学性
b. 研究数据的收集、记录和存储
c. 研究过程的偏离

1. 在和研究对象接触中的道德准则

a. 调查员的任务

代替研究团队收集数据的研究人员是整个项目的代表。有时调查员是项目参与者唯一直接接触到的人。他们的行为决定了与其接触的研究对象对整个研究印象的好坏。

调查员有责任确保研究对象是在知情同意基础上提供研究所需信息的。他们还必须确保信息的收集记录准确无误。否则，研究目标将无法完成。为了保证项目进行顺利，调查员必须认真遵守研究计划、项目实施手册和其它与参与者有关的研究程序进行操作。

b. 尊重待人的重要性

作为研究团队的一部分，每个研究人员都必须尊重：

•该研究项目的目标，
•该项目的负责人，
•该研究的参与者，
•参与该研究的社区，以及
•有助于实现项目目标而收集的数据。

科研项目最终将造福社会，但是研究课题组必须先要完整地完成该科研项目。每个研究团队成员在与社区参与者的接触中必须始终保持对对方的尊重。这包括尊重社区参与者的文化习惯、性别、年龄、社会地位、宗教信仰和其它独立的特征，以及他们提问的权利。科研项目参与者不承担必须帮助研究课题组的义务，所以如果研究课题组成员不尊重他们的选择权利，或不为他们提供最诚恳的态度和真实的信息，科研项目参与者将不会合作。

调查员以专业和尊重的方式收集数据会使每个参与者体会到研究项目的重要性。例如：

•无论参与者是否同意参与这项研究，始终礼貌待人。

•调查员需用清晰的声音提问。

•在调查表上整洁地记录信息。

•如果参加者提出了一个问题，请给与正确的回答。如果你不知道答案，但是可以从你的上级获得信息，如实告诉参与者你将在获得答案时告诉他们。

•向完成了研究程序的参与者表示感谢。

c. 自愿参与

没有任何人必须要参加一个研究项目。如果某项研究包括了知情同意过程，被邀请的潜在研究对象有权利拒绝听取有关研究的内容，并有权拒绝参加该研究。即使已经加入了研究项目，参与者仍然可以拒绝回答调查中的某些具体问题，拒绝提供某项标本或者参加某项测试，或者决定退出该研究项目。 征集参与者的研究小组成员必须负责确保参与者理解研究项目的目的和过程，完全自愿参加该研究，而不是因为害怕或其他原因被迫参加。

d. 知情同意

向可能被邀请参加研究项目的研究对象提供真实而准确的信息是进行有关人类研究的一个重要环节。知情同意是一个随着研究人员开始向参加者解释研究项目而进展的过程。知情同意的目的不仅是让参与者签署同意书和同意参加。事实上，知情同意的过程延续到整个研究过程；例如，调查员和参与者的每次接触都会有一个继续知情同意过程。如果参与者没有充分理解被要求做什么, 他们便没有真正的"自愿"加入该研究。调查小组需要提前决定信息传达的"充分" 程度和传达方法。负责知情同意的工作人员的任务是用参加者可以理解的语言，让他们了解这项研究的目的、过程、潜在的风险和研究的好处。所用的语言和沟通方式应该保证参加者能够理解。这种讨论应该给参与者足够的时间来提出问题，并思考和决定是否要参加研究。

有时，研究人员需要不时地向参与者确认他们是否已经理解研究内容和研究涉及的其它方面。负责获取知情同意的工作人员有时需要向参与者提问，以确定他们了解了些什么，理解的是否正确。另外，因为参与者可能看起来身体不舒服或有点困惑，但没有明确说出来，所以研究人员也应该留意观察参与者的肢体语言。如果观察到这些反应，研究人员应该告知他/她的上级人员来获得更多的指导。

e. 弱势人群

在征集某些人群参加项目时，需要特别注意和关心，因为他们的一些情况，使他们自己很难理解听到的内容，以及提供知情同意。例如，儿童需要额外的"保护"，同时他们的父母必须为他们作出某些决定。患有老年痴呆症的成人可能不明白你要求他们做什么。项目负责人准备的研究计划和实施手册应该告诉你如何接近这些人群和是否征集他们参加该项目。研究组成员必须非常小心地按照规则征集弱势人群参加研究，因为大多数时候弱势人群不能自行做出决定。如果该研究将征集不能自行做决定的参与者，必须有授权看护者或其他适当的代表人能代理他们决定。

f. 个人隐私

研究人员必须理解和尊重个人拥有的隐私权。即使文化中没有提倡或承认"隐私"的概念，尽可能地保护隐私仍然是很重要的。例如，如果没有被邀请就不能进入别人的家中，可能就是当地的一个习俗。研究组必须尊重这一习俗。或者，一个调查员在访问一个家庭时，可能会吸引好奇的旁观者。他们的出现可能会分散调查员和调查对象的注意力而对重要的数据收集过程产生不良影响。研究组必须预见到这个问题，并使它最小化。调查员要求保证个人隐私的举动，保证了对参与者的尊重，因为他们的家是家人生活的地方, 而不是公共场所。

研究组成员也必须尊重参与者的个人隐私，避免造成任何不必要的尴尬或不适。涉及敏感信息采访，应该在他人不能听到问题或答复的地方进行。体检不应该在他人可以看到的场所进

行。此外，有些话题是私人的敏感话题，如性生活、个人健康，或参与者不希望当众谈论的想法。

g. 个人信息的保护

如果研究参与者透露了参与者的个人信息，原本是高度保密的个人隐私就可能成为公开信息。也就是说，个人隐私可能受到侵犯。如果研究项目以外的人得到这些信息，参与者可能会遭遇尴尬、失业、法律问题，甚至来自社会各方面的伤害。研究人员应负责保护参与者避免受到这种伤害。

必须保证研究参与者所提供的个人信息的安全。任何无适当授权的人都不得看到或获取这些信息。如果这些信息是记录在纸上的，那么在锁进文件柜之前这些文件应该受到保护。只有经研究负责人授权的工作人员才能翻阅和处理这些信息。如果是电子信息，必须采取一切必要的防范措施以防止未经授权的人访问它。

有时，可使用随机调查数字标识数据，以保证无人知道数据是哪位参与者提供的。任何把数字与相关参与人的姓名联系起来的文件都必须被锁好以保持安全可靠。研究人员必须严格按照研究计划和实施手册的要求和内容保证研究数据的安全可靠。

h. 回应参与者的问题

一个调查员会遇到许多人向他/她询问关于研究的问题，这些人包括潜在的参与者、现有的参与者，以及好奇的旁观者。有些人不理解什么是“研究”，或不了解研究项目人员的任何情形。他们可能有各种各样的问题，其中一些可能和研究过程没有任何关系。 研究负责人需要事先培训调查员，让他们恰当回答人们可能提出的各种问题。因为，在日常的工作现场，是调查员代表了研究团队的所有人出现在参与者和整个社区面前。重要的是，调查员要尊重他人并尽其所能地回答问题。调查员必须有耐心，并准备回答任何参加者提出的问题。如果调查员知道答案，应当场回答。如果调查员自己不十分清楚答案的问题，则不应自行作答，因为给出错误的信息，比暂时无法回答会产生更大的负面作用。

如果你是调查员，当研究参与者提出了一个你不知道答案的问题，你应该告诉他你不确定，但你会把问题转达给上级，并将答案传达回给参与者。这一过程非常重要，因为一方面表明你尊重参与者，另一方面也确保你传递给参与者的答案会是准确的。当你觉得参与者没有问题了，你可以问“您还有其它问题吗？”，以确保所有得疑问都已经解决了。如果没有其它问题，那么你可以继续下一步。

2． 数据的完整性

a. 尊重研究的科学性

数据是“研究的成果”。确保收集、记录，和存储数据的准确性是至关重要的。科学家们将利用这些数据来回答在研究计划中提出的问题。如果数据是错误的，那么科学家们得到的答案将是错误的。由于这些结论和由此产生的行动是错误的，人们的生活可能因为错误的研究结果而受影响。所以，任何时候任何数据，正确收集、 记录并妥善贮存数据都是非常重要的。如果你犯了一个错误，必须要马上告诉你的上级, 以便让项目负责人或其他研究团队负责人知道。他们也许能解决这个问题，或判定有些数据可能无法使用。

b. 研究数据的收集、记录和存储

研究计划应该详细阐明该项目的目标，以及研究组将如何达到这些目标。数据的收集和记录的详细方法也应该包括在内，通常研究实施手册更加详细地列出这些步骤。调查员必须清楚理解究竟如何收集和记录数据。研究组负责人应当给调查员进行这方面的培训。如果调查员有任何问题，应当及时提出。事实上，如果调查员对具体怎样操作不清楚却不提问题，他们将无法确保数据是正确的。

一旦培训结束后，调查员开始数据收集工作。正确的数据收集需要按实施手册要求一步步完成并将数据完整无误地记录在调查表上。恰当的记录包括将答案用清晰、明确的方式写下来。

调查员必须记录真实而准确的信息。调查员不得记录未在调查表上列出的的额外信息。例如，如果调查表上没有“姓名”或“地址”这两栏，这些数据就不应该记录。调查表上不应该有虚构的信息.

恰当的数据存储指在数据传输到最终存储处的过程当中，采取各种安全防范措施。调查员不能把调查表放在可能会丢失、被盗或被研究组外的人看到的地方。调查表应由专门负责保管的人按事先定好的规范进行保存，以保护数据的保密性。如果数据是通过电子方法收集的，也应遵循同样的原则去维护其真实性、保密性和安全性。

c. 研究过程中的偏差

有时，一个调查员因为非个人原因而无法遵循原定的研究步骤，或者犯了错误。很重要的一点是必须将这些问题告知项目负责人，因为研究项目负责人可能有责任把这些问题报告给伦理审查委员会（IRB）。报告这些问题不要有羞耻感，这类问题会经常发生。调查员不上报这些问题是不对的，因为这可能意味着数据无效，或者某一个参与者在研究中有问题。同时这也意味着项目负责人无法将问题报告给伦理审查委员会。 称职的调查员应该将这些问题报告给上级，让其决定采取何种行动。

JHSPH HUMAN SUBJECTS RESEARCH ETHICS
FIELD TRAINING GUIDE

This guide is intended to be used as a tool for training individuals who will be "engaged" in some aspect of a human subject research interaction or intervention. It is directed, in particular, to Johns Hopkins principal investigators who are responsible for training of study team members who will (1) obtain informed consent from research participants, or (2) collect data from human participants through individual or focus group interviews, testing, physical measurements, or other procedures involving direct contact, hereafter called a "data collector". The content and language level of this guide is specifically worded to help the investigator convey basic research principles and behavior that accords with those principles to data collectors. We encourage users who translate the document into local languages to submit those translations (with certifications of the translator's qualifications) to our office (irboffice@jhsph.edu) so we can make them available to other investigators.

TABLE OF CONTENTS

1. Ethical Interaction with Human Participants
    - a. Role of the Data Collector
    - b. Importance of Respect
    - c. Voluntary Participation
    - d. Informed Consent
    - e. Vulnerable Populations
    - f. Personal Privacy
    - g. Protection of Personal Information
    - h. Response to Participant Questions

2. Data Integrity
    - a. Respect for the Science of the Study
    - b. Collecting, Recording, and Storing Study Data
    - c. Deviations from Study Procedures

1. **Ethical Interaction with Human Participants**

a. Role of the Data Collector

The person who collects information on behalf of a research team is an "ambassador" for the study. The data collector is sometimes the only person on the study team with whom a research participant will come into contact.

People who come into contact with that person will have a good impression of the study or a bad impression of the study, depending on how the data collector presents him- or herself.

The data collector has the responsibility for making sure that the information collected for the study comes from individuals who understand what they are agreeing to do. In addition, the data collector must ensure that the information collected and recorded is accurate and protected from loss. Otherwise, the study objectives will not be achieved. To be successful, the data collector must carefully follow the research plan, study operations manual and other procedures involving contact with human participants.

b. Respect

Each person who is part of the research team must show respect for:

- the goals of the research project,
- the leaders of the project,
- the individual study participant,
- the participant community and
- the data collected that will help achieve project objectives.

The scientific project has the potential to benefit the community that will be studied, but only if the research team is able to complete all the parts of the study.
Each individual research team member must conduct all interactions with members of the participant community with respect. This includes respecting the participant community's culture, gender, age, social status, religion and other characteristics that make people different from each other, as well as their right to ask questions. The individual does not have to help the research team by participating in the study, and they will not do so if the research team is not respectful of the participant's right to say "yes" or "no", or to receive honest and true information from all team members

The data collector conveys to each participant the importance of the study purpose by collecting data in a professional and respectful manner. For example:

- Always be polite to the participant whether or not she or he agrees to participate in the study.
- If the data collector is asking questions, ask them in a clear voice.
- Record the information neatly on the data collection sheets.
- If the participant asks a question, provide an answer that is correct. If you do not know the answer, and it is possible for you to obtain the information from your supervisor, tell the participant that you will obtain an answer to their question and that you will let them know what you find out.
- Thank the participants once they have completed the study procedures.

c. Voluntary Participation
No individual person is <u>required</u> to participate in a research project. If the study includes an informed consent process, then each person approached by a study team member has the right to refuse to hear about the study, and the right to refuse to join the study. Even if a person joins the study, he or she may refuse to answer specific questions in a survey or questionnaire, refuse to give a specimen or refuse to take a test, and may decide to withdraw from a study at any time.
The research team member who obtains informed consent from participants is responsible for ensuring that the individual understands what the study is about and truly agrees to join the study and is not joining because they are afraid not to, or feel forced to join.

d. Informed Consent
Providing correct, factual information to persons being approached to join a study is an essential part of human subjects research. Informed consent is an ongoing process that begins with the research team member explaining the study to the participant. Informed consent does not end with the participant signing the consent form and agreeing to be in the study. The process of informed consent continues throughout the study; for example, it can occur each time a data

collector and participant interact. There is no true “consent” to join a study if a person does not adequately understand what is being asked of them. The investigative team determines ahead of time what is “adequate” and how that information should be conveyed. The job of the person conducting informed consent is to present information about the study to the potential participant in language that s/he can understand, and in a way that reveals the study purpose, procedures, potential risks and benefits of the study. The language and style of communication should enable a participant to understand. That discussion should give the participant enough time to ask questions and to think about the decision whether or not to join the study. Sometimes it is important to check in with the participant from time to time to make sure that the participant continues to understand what the study is about, or what it involves. The study team member who is obtaining consent may have to ask the participant questions to see what the participant has learned, and whether the participant has the correct understanding. The research team member should also be aware of the participant’s body language, as the participant may look physically uncomfortable or confused, but not say so. If these responses are observed, the study team member should notify his/her supervisor for further guidance.

e. Vulnerable Populations

Some people need extra attention and care when approached to participate in a research project because they have conditions that make it difficult for them to understand what is being told to them and to provide informed consent. For example, children need extra “protection” and it is important that their parents make certain decisions for them. Adults who have dementia may not understand what you are asking them to do. The research plan and the operations manual, developed by the investigators, should tell you how to approach these individuals, and if they are to be included in the study. Research team members must be very careful to follow the rules when enrolling vulnerable populations because most of the time they cannot make decisions for themselves. If the study will include people who cannot make decisions for themselves, an authorized caregiver or other proper representative must be available to decide for them.

f. Personal Privacy

Individuals have a right to privacy that the research team must understand and respect. Even if the culture does not promote or generally give recognition to the concept of “privacy”, it is important that the right to privacy be protected to every possible extent. For example, it may be the custom that no one enters another person’s home without being invited. The study team must honor this custom. Or, the visit of a data collector to a home may attract curious onlookers, whose presence may be undesirable and will distract the data collector and participant from focusing on the important process of data collection. The study team must anticipate this problem and minimize it. When a data collector requests privacy, that action assures the respondent that the data collector respects that the home is where the family lives; it is not a public space.

Research team members must also respect the participant’s personal privacy by not causing them any unnecessary personal embarrassment or discomfort. Interviews involving sensitive information should take place where other people cannot hear the questions or responses. Physical examinations should not occur where other people can watch. Also, there are certain things that are considered to be “private”, such as sexual activity, personal health, or thoughts that one might not want to talk about in public.

g. Protection of Personal Information
When a study participant discloses personal information about him or herself to a data collector, that participant is at risk of having highly confidential information become “public”. That is, s/he risks losing confidentiality. The risk is that if someone outside the study learns about the private information, bad things could happen to the participant, like embarrassment, loss of employment, legal problems, or social damage. The research team is responsible for protecting the participant from this kind of injury.

After a study participant has provided information, that personal information must be kept safe. No one without the proper authority should see or have access to the information. If the information is written on paper, then that paper should be protected until it is locked up in a cabinet. It must only be seen and processed by study staff who are authorized by the study investigators to handle the information. If the information is electronic, then all necessary precautions should be taken to make sure that no unauthorized person can access it. Sometimes a random study number is used to identify the data so that no one will know which participant the data came from. Any document that links a number with the name of the person it is assigned to must be locked up and kept safe and secure. The research plan and operations manual must be followed to make sure that the study data are protected exactly as prescribed in the manuals.

h. Response to Participant’s Questions
A data collector will meet many people, including prospective participants, existing study participants and curious onlookers not involved in the study, who will have questions about the study. Some people will not understand what “research” is, or will not know anything about the researchers who are leading this project. They may have all sorts of questions, some of which may not have anything to do with the study procedures at all.

Investigators will train data collectors to address the many concerns that are likely to be expressed by people. This is because, in the “field” on a day to day basis, it is the data collector who represents the study when talking with possible participants and the community at large. It is important that the data collector show proper respect to all individuals and do one’s best to address concerns. A data collector must be patient and answer any question that a participant asks, so long as s/he knows the answer! A data collector should never answer questions for which the answers are not clearly known, because giving wrong information can be worse than giving no information, at least temporarily. If you are a data collector and a participant asks a question and you are not sure of the answer, here’s what should happen: you should tell the person that you do not have a confident answer to the question; that you will ask the study supervisor the question; and that you will pass that answer on to the participant. This is very important because it shows respect to the participant and it makes sure that the information you pass on to the participant is accurate. When you think the participant has no more questions, you may ask, “Do you have any other questions?” to make sure that all questions have been addressed. If there are no more questions, then you may proceed.

2. Data Integrity
a. Respect for the Science of the Study
Data are the “product” of research. It is very important that the information collected, recorded, and stored by the data collectors is correct. Scientists will use these data to answer the research questions identified in the research plan. If the data are wrong, then the answers that

the scientists produce will also be wrong. People whose lives may be affected by the results of the study may be put at risk, because the answers and actions that follow will be wrong. So it is very important that all data at all times are collected properly, recorded properly, and stored properly. If you make a mistake doing any of these things, it is important that you tell your supervisor right away so that the investigators or research team leaders know about it. They may be able to fix the problem, or the will know that some data may not be usable.

b. Collecting, Recording, and Storing Study Data
The research plan spells out the project objectives and how the research team will reach those objectives. The details of data collection and recording study data are included, and usually the study operations manual goes into more detail about how those jobs will be done. The data collector must understand exactly how the data should be collected, and how they are recorded. The research team leaders will train the data collectors on this process. If the data collectors have any questions, they must not be afraid to ask them. In truth, if the data collectors do not ask questions when they are unsure of how things should be done, they will not be able to make sure that the data are correct.

Once the training is complete, the data collectors begin their job. Good data collection means following the instructions and accurately completing the data collection sheet. Proper recording includes making sure that the answers to questions are written in a legible and clear way. The data collector must record the information with honesty and accuracy. Extra information that is not identified in the data collection sheet should not be included. For example, if there is no space for “name” or “address”, then these data should not be recorded on the data collection sheet. No information should be “made up” and recorded on the data collection sheet.

Proper storing of data means that all safety precautions should be taken while transporting the data to the ultimate storage place. Data collectors should not put the collection sheets down where they might be lost, stolen, or read by someone outside the research team. The data collection sheets should be given to the person responsible for storage, and that person should follow all the instructions to protect data confidentiality. If data are collected electronically, the same principles and rules of honesty, protection and care must be followed.

c. Deviations from Study Procedures
Sometimes a data collector is not able to follow study procedures through no fault of his or her own, and sometimes a data collector may make a mistake. It is very important to let the research leaders know about these problems because the research leaders have the responsibility to report these kinds of problems to the reviewing Institutional Review Board (IRB). There is no shame in reporting these kinds of problems. They happen all the time. It is not good, however, if the data collector fails to report these problems because it could mean that the data are not good, or that a participant has a problem with the study. It also means that the study supervisor will not be able to complete the report to the IRB.

A good data collector will communicate these issues to his or her supervisor and let that person decide what action to take.
