# Supplementary material for: A Field Training Guide for Human Subjects Research Ethics
Source: PLoS Med. 2010 Oct 5;7(10):e1000349. doi: 10.1371/journal.pmed.1000349 (PMC2950122; doi:10.1371/journal.pmed.1000349)
Supplement: Text S4 — Thai translation of JHSPH Field Training Guide courtesy of David Celentano and Louise Walshe. (0.17 MB PDF) [file pmed.1000349.s004.pdf]

หลักจริยธรรมการวิจัยในอาสาสมัครที่เป็นมนุษย์
ของวิทยาลัยสาธารณสุขบลูมเบิร์ก มหาวิทยาลัยจอห์นส์ ฮ้อพกิ้นส์ (JHSPH)
แนวทางการฝึกอบรมภาคสนาม

แนวปฏิบัตินี้มีจุดประสงค์เพื่อใช้เป็นเครื่องมือสำหรับการฝึกอบรมบุคลากรที่จะ "มีส่วนเกี่ยวข้อง" ในบางส่วนของการปฏิสัมพันธ์หรือการแทรกแซงกับอาสาสมัครวิจัยที่เป็นมนุษย์ มุ่งหมายให้ผู้วิจัยหลัก(PI)ของมหาวิทยาลัยจอห์นส์ ฮ้อพกิ้นส์ ผู้มีหน้าที่รับผิดชอบในการฝึกอบรมแก่เจ้าหน้าที่ในทีมวิจัย ผู้ซึ่งจะ (1) ขอความยินยอมโดยได้รับการบอกกล่าวจากอาสาสมัครวิจัย หรือ (2) เก็บข้อมูลของอาสาสมัคร โดยจากทั้งการสัมภาษณ์เป็นรายบุคคลหรือแบบกลุ่มเฉพาะ จากการทดสอบ จากการวัดทางกายภาพ หรือกระบวนการอื่นๆที่เป็นการติดต่อโดยตรง ซึ่งต่อจากนี้จะเรียกว่า "ผู้เก็บข้อมูล" การเขียนเนื้อหาและระดับของภาษาที่ใช้ในแนวปฏิบัตินี้ เป็นไปเพื่อช่วยผู้วิจัยให้สามารถนำเอาหลักการวิจัยเบื้องต้นและพฤติกรรมที่สอดคล้องกับหลักการวิจัยนี้ส่งต่อให้กับผู้เก็บข้อมูล เราสนับสนุนให้ผู้ใช้แปลเอกสารเป็นภาษาท้องถิ่นต่างๆ และจะยินดียิ่งหากได้ส่งฉบับแปลมายังสำนักงานของเรา (irboffice@jhsph.edu) เพื่อเผยแพร่ต่อไป

รายการเนื้อหา

1. จรรยาบรรณในการปฏิบัติต่ออาสาสมัครที่เป็นมนุษย์
    ก. บทบาทของผู้เก็บข้อมูล
    ข. ความสำคัญของการเคารพ
    ค. การเข้าร่วมโดยสมัครใจ
    ง. การขอคำยินยอมโดยได้รับการบอกกล่าว
    จ. ประชากรที่อ่อนด้อย
    ฉ. ความเป็นส่วนตัว
    ช. การปกป้องคุ้มครองข้อมูลส่วนตัว
    ซ. การตอบคำถามของอาสาสมัคร

2. ความน่าเชื่อถือของข้อมูล
    ก. เคารพต่อวิธีการของการศึกษา
    ข. การเก็บข้อมูล การบันทึกและการเก็บรักษาข้อมูลการศึกษา
    ค. การเบี่ยงเบนออกจากวิธีปฏิบัติในการศึกษา

1. จรรยาบรรณในการปฏิบัติต่ออาสาสมัครที่เป็นมนุษย์

ก. บทบาทของผู้เก็บข้อมูล

บุคคลผู้เก็บข้อมูลเพื่อประโยชน์ของทีมผู้วิจัยเป็นดั่งตัวแทนของการวิจัย เพราะผู้เก็บข้อมูลบางครั้งเป็นบุคคลเพียงคนเดียวของทีมวิจัยผู้ที่อาสาสมัครจะสามารถมีปฏิสัมพันธ์ด้วย บุคคลที่พูดคุยด้วยจะมีความรู้สึกที่ดีหรือไม่ดีต่อการศึกษา ขึ้นอยู่กับว่าผู้เก็บข้อมูลจะแนะนำตนเองอย่างไร

ผู้เก็บข้อมูลมีหน้าที่ทำให้เกิดความมั่นใจว่าข้อมูลที่ถูกเก็บมาเพื่อการศึกษา มาจากผู้ที่เข้าใจสิ่งที่เขาตกลงจะทำ นอกจากนี้ผู้เก็บข้อมูลต้องมั่นใจว่าข้อมูลที่ได้เก็บและบันทึกมามีความถูกต้องและมีการป้องกันจากการสูญหาย มิฉะนั้นแล้ววัตถุประสงค์ของการวิจัยจะไม่บรรลุผลสำเร็จ และเพื่อให้บรรลุผลสำเร็จ ผู้เก็บข้อมูลจะต้องปฏิบัติตามแผนการวิจัย คู่มือปฏิบัติการวิจัย และกระบวนการอื่นๆที่เกี่ยวข้องกับการติดต่ออาสาสมัคร อย่างรอบคอบ

ข. ความสำคัญของการเคารพ

เจ้าหน้าที่แต่ละคนที่เป็นส่วนหนึ่งของทีมวิจัยต้องเคารพต่อ:

- เป้าหมายของโครงการวิจัย
- หัวหน้าโครงการวิจัย
- บุคคลผู้เป็นอาสาสมัครวิจัย
- ชุมชนที่เข้าร่วม และ
- ข้อมูลที่ถูกเก็บ เพื่อช่วยให้บรรลุวัตถุประสงค์ของโครงการ

โครงการทางวิทยาศาสตร์มีศักยภาพในการให้ประโยชน์แก่ชุมชนที่จะได้ทำการศึกษา หากทีมวิจัยสามารถทำให้สำเร็จได้ในทุกส่วนของการวิจัย

เจ้าหน้าที่ทีมวิจัยแต่ละคนต้องปฏิบัติต่อสมาชิกทั้งหมดในชุมชนที่เข้าร่วมด้วยความเคารพ รวมถึงการเคารพต่อวัฒนธรรม เพศสภาวะ (Gender) อายุ สถานภาพทางสังคม ศาสนา และลักษณะพิเศษอื่นๆของชุมชนที่เข้าร่วม ที่ทำให้ผู้คนมีความแตกต่างจากที่อื่นๆ รวมถึงสิทธิในการถามคำถาม อาสาสมัครไม่จำเป็นต้องช่วยทีมวิจัยโดยการเข้าร่วมการวิจัย และอาจไม่เข้าร่วมหากทีมวิจัยไม่ให้

ความเคารพต่อสิทธิของอาสาสมัครในการตัดสินใจว่าจะ “เข้าร่วม” หรือ “ไม่เข้าร่วม” หรือให้ได้รับข้อมูลที่ถูกต้องและเป็นจริงจากสมาชิกในทีมทั้งหมด

ผู้เก็บข้อมูลแสดงให้อาสาสมัครแต่ละคนเห็นถึงความสำคัญของจุดประสงค์ของการวิจัยโดยการเก็บข้อมูลด้วยวิธีที่น่าเชื่อถือและเป็นมืออาชีพ ตัวอย่างเช่น:

- สุภาพต่ออาสาสมัครเสมอ แม้ว่าอาสาสมัครจะตกลงเข้าร่วมในการวิจัยหรือไม่ก็ตาม
- ถ้าผู้เก็บข้อมูลถามคำถาม ให้ถามด้วยน้ำเสียงที่ชัดเจน
- บันทึกข้อมูลให้เรียบร้อยในแผ่นบันทึกข้อมูล
- ถ้าอาสาสมัครมีคำถามให้ตอบคำถามที่ถูกต้อง หากไม่ทราบคำตอบ และเป็นไปได้ที่จะได้รับคำตอบนั้นจากหัวหน้าของท่าน ให้บอกอาสาสมัครว่าท่านจะหาคำตอบมาและจะแจ้งให้ทราบ
- ขอบคุณอาสาสมัครเมื่อเสร็จสิ้นกระบวนการศึกษา

ค. การเข้าร่วมโดยสมัครใจ

ไม่มีผู้ใดถูกกำหนดให้เข้าร่วมในโครงการวิจัย หากการวิจัยประกอบด้วยกระบวนการขอคำยินยอมโดยได้รับการบอกกล่าว อาสาสมัครแต่ละคนที่เจ้าหน้าที่ทีมวิจัยเข้าหา มีสิทธิในการปฏิเสธที่จะรับฟังเกี่ยวกับการวิจัย และมีสิทธิปฏิเสธการเข้าร่วมการวิจัย และถึงแม้เข้าร่วมการวิจัยแล้วก็อาจปฏิเสธในการตอบคำถามที่เฉพาะเจาะจงในแบบสำรวจหรือในแบบสอบถาม ปฏิเสธการให้ตัวอย่างสิ่งส่งตรวจ หรือปฏิเสธที่จะทำการทดสอบ และอาจตัดสินใจถอนตัวออกจากการศึกษาได้ทุกเวลา

เจ้าหน้าที่ทีมวิจัยที่ได้รับคำยินยอมจากอาสาสมัคร มีหน้าที่ทำให้มั่นใจว่าอาสาสมัครเข้าใจว่าการวิจัยนั้นเกี่ยวกับอะไร และตกลงเข้าร่วมการวิจัยจริงๆ และไม่ใช่เข้าร่วมเพราะว่าอาสาสมัครกลัวหรือรู้สึกว่าถูกบังคับให้เข้าร่วม

ง. การขอคำยินยอมโดยได้รับการบอกกล่าว

การให้ข้อมูลที่ถูกต้อง เป็นจริงแก่บุคคลที่ถูกทาบทามให้เข้าร่วมโครงการซึ่งเป็นส่วนที่มีความสำคัญของการวิจัยในอาสาสมัครที่เป็นมนุษย์ การขอคำยินยอมโดยได้รับการบอกกล่าวเป็นกระบวนการต่อเนื่องที่เริ่มต้นโดยเจ้าหน้าที่ทีมวิจัยอธิบายการศึกษาแก่อาสาสมัคร การขอคำ

ยินยอมโดยได้รับการบอกกล่าวไม่เสร็จสิ้นเพียงแค่อาสาสมัครลงชื่อในแบบฟอร์มการขอคำยินยอมและตกลงเข้าร่วมการวิจัยเท่านั้น แต่จะดำเนินต่อไปตลอดการศึกษา ตัวอย่างเช่น อาจเกิดขึ้นได้ในแต่ละครั้งที่ผู้เก็บข้อมูลและอาสาสมัครมีปฏิสัมพันธ์กัน จะถือว่า “ความยินยอม” นั้นยังไม่ใช่ความยินยอมที่แท้จริง หากอาสาสมัครไม่ได้มีความเข้าใจอย่างเพียงพอในสิ่งที่กำลังถูกขอ ทีมวิจัยกำหนดล่วงหน้าว่าเวลาเท่าไหร่คือ “เพียงพอ” และจะให้ข้อมูลนั้นอย่างไร งานของผู้ดำเนินการขอคำยินยอมคือ ชี้แจงข้อมูลเกี่ยวกับการศึกษาแก่อาสาสมัครในภาษาที่ทำให้อาสาสมัครเข้าใจ และแสดงถึงจุดประสงค์ของการศึกษา กระบวนการ ความเสี่ยงที่อาจมี และประโยชน์ของการศึกษา ภาษาและรูปแบบของการสื่อสารต้องเป็นไปเพื่อให้อาสาสมัครเข้าใจได้ ต้องให้เวลาอาสาสมัครอย่างเพียงพอเพื่อถามคำถามและตัดสินใจว่าจะเข้าร่วมการวิจัยหรือไม่

บางครั้งเป็นสิ่งสำคัญที่จะต้องเน้นย้ำอาสาสมัครทุกครั้งตามความเหมาะสมเพื่อให้มั่นใจว่าอาสาสมัครเข้าใจอย่างต่อเนื่องเกี่ยวกับการวิจัยหรืออะไรที่เกี่ยวข้อง เจ้าหน้าที่ทีมวิจัยที่ได้รับคำยินยอมอาจถามคำถามอาสาสมัครเพื่อทดสอบว่าอาสาสมัครทราบและเข้าใจถูกต้องหรือไม่ เจ้าหน้าที่ทีมวิจัยต้องสังเกตภาษากายของอาสาสมัครด้วย ซึ่งอาสาสมัครอาจมีความไม่สบายใจหรือมีความสับสนแต่ไม่พูดออกมา หากสังเกตได้ว่ามีปฏิกิริยาเหล่านี้ เจ้าหน้าที่ทีมวิจัยควรแจ้งผู้ดูแลของตนเพื่อขอคำแนะนำต่อไป

จ. ประชากรที่อ่อนด้อย

บางคนต้องการการใส่ใจและดูแลเป็นพิเศษในการทาบทามให้เข้าร่วมโครงการวิจัย เพราะบุคคลเหล่านั้นอยู่ในสภาพที่ยากต่อการเข้าใจในสิ่งที่กำลังบอกแก่ตน และยากต่อการให้คำยินยอมโดยได้รับการบอกกล่าว ตัวอย่างเช่น เด็กต้องการ “การคุ้มครอง” เป็นพิเศษและเป็นสิ่งสำคัญที่พ่อแม่ของเด็กจะตัดสินใจบางอย่างแทนเด็ก หรือผู้ใหญ่ที่มีปัญหาทางด้านสมองอาจไม่เข้าใจในสิ่งที่ท่านขอให้เขาทำ โครงร่างการวิจัยและคู่มือวิธีปฏิบัติที่พัฒนาขึ้นมาโดยผู้วิจัยจะบอกถึงวิธีการที่จะเข้าถึงพวกเขาเหล่านี้ และหากพวกเขาได้เข้าร่วมในโครงการวิจัย เจ้าหน้าที่ทีมวิจัยต้องระมัดระวังอย่างมากโดยการปฏิบัติตามระเบียบต่างๆเมื่อรับอาสาสมัครที่อยู่ในกลุ่มประชากรที่อ่อนด้อย เพราะตลอดเวลาโดยส่วนมากที่พวกเขาจะไม่สามารถตัดสินใจทำอะไรได้ด้วยตัวเอง

ฉ. ความเป็นส่วนตัว

บุคคลมีสิทธิในความเป็นส่วนตัว ซึ่งเจ้าหน้าที่ทีมวิจัยต้องเข้าใจและให้ความเคารพ แม้ว่าโดยสถานการณ์แล้วจะไม่ส่งเสริมหรือรับรองให้เป็นไปตามหลักการโดยทั่วไปของ “ความเป็น

ส่วนตัว" ได้ เป็นสิ่งสำคัญที่สิทธิความเป็นส่วนตัวต้องได้รับการปกป้องในทุกๆขอบเขตที่เป็นไปได้ ตัวอย่างเช่น อาจมีธรรมเนียมที่ว่าจะไม่มีการเข้าบ้านคนอื่นโดยไม่ได้รับการเชื้อเชิญ ในอีกด้านหนึ่งการเข้าพบของผู้เก็บข้อมูลที่บ้านอาจเป็นการดึงดูดผู้พบเห็นที่มีความอยากรู้อยากเห็น ซึ่งก็ไม่ควรส่งเสริมในท่าทีของผู้คนเหล่านั้น นั่นเพราะเพื่อปกป้องทั้งอาสาสมัครและเพื่ออำนวยให้ผู้เก็บข้อมูลและอาสาสมัครมีสมาธิกับขั้นตอนที่สำคัญของการเก็บข้อมูล การร้องขอความเป็นส่วนตัวทำให้อาสาสมัครเชื่อมั่นได้อีกว่าผู้เก็บข้อมูลได้ให้ความเคารพว่าบ้านคือสถานที่อยู่อาศัยของครอบครัว ไม่ใช่พื้นที่สาธารณะ

เจ้าหน้าที่ทีมวิจัยยังต้องให้ความเคารพกับความเป็นส่วนตัวของอาสาสมัคร โดยไม่ทำอะไรที่จะทำให้เขารู้สึกอับอายหรืออึดอัดโดยไม่จำเป็น การสัมภาษณ์เกี่ยวกับข้อมูลที่มีความอ่อนไหว จะต้องทำในสถานที่ที่ผู้อื่นไม่สามารถได้ยินคำถามหรือคำตอบ การตรวจร่างกายไม่ควรทำในสถานที่ที่บุคคลอื่นสามารถมองเห็นได้ และยังมีบางอย่างที่พิจารณาได้ว่าเป็นเรื่องส่วนตัว เช่น กิจกรรมทางเพศ สุขภาพส่วนตัว หรือความคิดเห็นต่างๆที่บุคคลอาจไม่ต้องการพูดในที่สาธารณะ

ช. การปกป้องคุ้มครองข้อมูลส่วนตัว

เมื่ออาสาสมัครเปิดเผยข้อมูลส่วนตัวของตนแก่ผู้เก็บข้อมูล นั่นหมายความว่าอาสาสมัครกำลังเสี่ยงว่าข้อมูลของตนจะถูก "เปิดเผย" ทำให้เสี่ยงต่อการสูญเสียการเป็นความลับ ความเสี่ยงนั้นคือ หากบุคคลใดนอกเหนือจากการวิจัยรับรู้ถึงข้อมูลส่วนตัวนั้น ผลเสียย่อมเกิดขึ้นกับอาสาสมัคร เช่น ความอับอาย การถูกเลิกจ้าง ปัญหากับเจ้าหน้าที่ตำรวจ หรือผลกระทบทางสังคม ทีมวิจัยมีความรับผิดชอบในการปกป้องคุ้มครองอาสาสมัครจากความเสียหายเหล่านี้

หลังจากที่อาสาสมัครได้ให้ข้อมูลแล้ว ข้อมูลส่วนตัวต้องเก็บไว้อย่างปลอดภัย บุคคลที่ไม่มีอำนาจเหมาะสมไม่สามารถดู หรือเข้าถึงข้อมูลนั้นได้ หากข้อมูลนั้นบันทึกไว้ในกระดาษ กระดาษเหล่านั้นต้องได้รับการปกป้องจนกว่าจะได้จัดเก็บไว้ในตู้ที่มีกุญแจล็อค เจ้าหน้าที่โครงการที่ได้รับมอบหมายจากผู้วิจัยในการจัดการข้อมูลเท่านั้นที่จะสามารถเห็นและดำเนินการกับข้อมูลนั้นได้ แต่หากข้อมูลบันทึกไว้ในระบบอิเล็คทรอนิคส์ จะต้องมีการป้องกันทุกวิถีทางเท่าที่จำเป็น เพื่อให้มั่นใจว่าจะไม่มีผู้ใดสามารถเข้าถึงได้โดยไม่ได้รับอนุญาต

บางครั้งมีการใช้ตัวเลขเลือกสุ่มเพื่อระบุที่มาของข้อมูล ซึ่งจะไม่มีใครทราบว่าข้อมูลนั้นมาจากอาสาสมัครคนใด เอกสารใดๆที่จะเชื่อมโยงกับตัวเลขและชื่อของบุคคลต้องได้รับการเก็บไว้ในตู้ที่

มีกุญแจล็อคและอยู่ในสถานที่ที่ปลอดภัย โดยการปฏิบัติตามโครงร่างการวิจัยและคู่มือวิธีปฏิบัติ เพื่อทำให้มั่นใจว่าข้อมูลในการวิจัยนั้นได้รับการปกป้องอย่างแท้จริงตามที่ได้กำหนดไว้ในคู่มือ

ซ. การตอบคำถามของอาสาสมัคร

หลายๆคน รวมถึง บุคคลที่คาดหวังว่าจะเข้าร่วม อาสาสมัครปัจจุบัน และผู้คนที่อยากรู้อยากเห็น แต่ไม่เกี่ยวข้องกับการศึกษาที่ผู้เก็บข้อมูลพบเจอจะมีคำถามเกี่ยวกับการศึกษา บางคนไม่เข้าใจว่า "วิจัย" คืออะไร หรือไม่ทราบอะไรเลยเกี่ยวกับผู้วิจัยที่เป็นหัวหน้าโครงการ พวกเขาอาจจะมีคำถาม มากมาย บางคำถามอาจไม่เกี่ยวข้องกับกระบวนการในการศึกษาเลย

ผู้วิจัยจะอบรมผู้เก็บข้อมูลเพื่อเตรียมรับกับข้อกังวลหลายๆอย่างที่คาดการณ์ได้ และมีแนวโน้มว่าจะ ถูกถามจากผู้คน เนื่องจากว่าในการทำงาน "ภาคสนาม" ที่ในกิจวัตรทุกๆวัน ผู้เก็บข้อมูลซึ่งเป็น ตัวแทนของการวิจัย เมื่อต้องพูดคุยกับผู้ที่คาดว่าจะเป็นอาสาสมัคร และชุมชมโดยรวม เป็นความ จำเป็นที่ผู้เก็บข้อมูลต้องแสดงความเคารพอย่างเหมาะสมกับทุกคน และทำทุกอย่างเพื่อพูดคุย เกี่ยวกับความกังวลของพวกเขาให้ได้อย่างดีที่สุด ผู้เก็บข้อมูลควรมีความอดทน และตอบทุกคำถาม ที่อาสาสมัครมี จนกว่าผู้เก็บข้อมูลทราบคำตอบทุก ผู้เก็บข้อมูลไม่ควรตอบคำถามที่ไม่ทราบอย่าง ชัดเจน เพราะการให้ข้อมูลที่ผิดจะส่งผลเสียมากกว่าการไม่ได้ให้ข้อมูล ซึ่งอย่างน้อยก็เป็นเพียงชั่ว ระยะเวลาหนึ่ง ในสถานการณ์เช่นนี้ บอกกับบุคคลนั้นว่าท่านไม่มั่นใจในคำตอบ ซึ่งท่านจะได้ถาม ผู้ดูแลและจะนำคำตอบมาบอกแก่อาสาสมัคร เป็นเรื่องที่สำคัญมากเพราะจะแสดงถึงการเคารพต่อ อาสาสมัคร และทำให้มั่นใจได้ว่า ข้อมูลที่ส่งต่อให้กับอาสาสมัครนั้นถูกต้อง เมื่อท่านคิดว่า อาสาสมัครคงไม่มีคำถามใดๆแล้ว ท่านอาจจะถามว่า "คุณมีคำถามอะไรอื่นๆอีกหรือไม่?" เพื่อให้ มั่นใจว่าทุกคำถามได้รับการพูดคุยแล้ว หากว่าไม่มีคำถามใดๆอีก ท่านก็สามารถดำเนินการต่อไป ได้

2. ความน่าเชื่อถือของข้อมูล

ก. เคารพต่อวิธีของการศึกษา

ข้อมูลคือ "ผล" ของการวิจัย สิ่งนี้มีความสำคัญอย่างมากที่ข้อมูลซึ่งได้มา บันทึกและเก็บรักษาไว้ โดยผู้เก็บข้อมูลนั้นจะต้องมีความถูกต้อง นักวิทยาศาสตร์จะใช้ข้อมูลเหล่านี้เพื่อตอบคำถามการวิจัย ที่ระบุไว้ในโครงร่างการวิจัย หากข้อมูลนั้นผิด คำตอบที่นักวิทยาศาสตร์นั้นสรุปขึ้นมาก็ย่อมผิดไป ด้วย ชีวิตของผู้คนอาจได้รับผลกระทบจากผลของการศึกษา อาจตกอยู่ในความเสี่ยง เพราะว่า

คำตอบนั้นผิดและการกระทำที่ตามมาก็จะผิดด้วย ดังนั้นจึงมีความสำคัญอย่างยิ่งที่ข้อมูลทั้งหมดจะต้องมีการเก็บอย่างเหมาะสม บันทึกอย่างเหมาะสม และเก็บรักษาไว้อย่างเหมาะสมอยู่ตลอดเวลา หากเกิดข้อผิดพลาดที่ท่านทำไว้กับสิ่งเหล่านี้ จำเป็นอย่างยิ่งที่ท่านจะต้องบอกกับผู้ดูแลโดยทันที เพื่อว่าผู้วิจัยหรือหัวหน้าทีมวิจัยจะได้รับทราบ และอาจจะแก้ไขปัญหา หรือไม่จะได้ทราบว่าข้อมูลบางชิ้นไม่สามารถนำมาใช้ได้

ข. การเก็บข้อมูล การบันทึกและการเก็บรักษาข้อมูลการวิจัย

โครงร่างการวิจัยได้บอกไว้อย่างชัดเจนถึงวัตถุประสงค์ของการศึกษา และวิธีการที่ทีมวิจัยจะบรรลุถึงวัตถุประสงค์นั้น รายละเอียดของการเก็บข้อมูล และบันทึกข้อมูลการวิจัย จะรวมไว้อยู่ในโครงร่างการวิจัยนี้ และโดยทั่วไปคู่มือวิธีปฏิบัติจะมีข้อมูลที่เป็นรายละเอียดในวิธีดำเนินการให้สำเร็จลุล่วง ผู้เก็บข้อมูลต้องเข้าใจอย่างถ่องแท้ว่าควรจะเก็บข้อมูลอย่างไร และควรจะบันทึกอย่างไร หัวหน้าทีมวิจัยควรต้องอบรมกระบวนการเหล่านี้ให้กับผู้เก็บข้อมูล หากผู้เก็บข้อมูลมีปัญหาใดๆ ก็ไม่ควรเกรงใจที่จะถาม ในความเป็นจริง หากผู้เก็บข้อมูลไม่ถามคำถามขณะที่พวกเขาไม่แน่ใจว่าควรจะทำสิ่งนั้นสิ่งนี้อย่างไร พวกเขาจะไม่สามารถรับประกันได้ว่าข้อมูลที่ได้มานั้นจะถูกต้อง

หลังจากที่การอบรมสิ้นสุดลง ผู้เก็บข้อมูลจึงสามารถเริ่มงานได้ การเก็บข้อมูลที่ดีหมายถึงการปฏิบัติตามข้อแนะนำและการกรอกข้อมูลที่สมบูรณ์ ถูกต้องในแผ่นบันทึกข้อมูล การบันทึกข้อมูลอย่างเหมาะสม รวมถึงการทำให้มั่นใจว่าคำตอบได้กรอกด้วยลายมือที่อ่านได้และไม่รกรุงรัง ผู้เก็บข้อมูลต้องบันทึกข้อมูลด้วยความซื่อสัตย์และมีความถูกต้อง ข้อมูลอื่นๆเพิ่มเติมที่ไม่ได้ระบุไว้ในแผ่นบันทึกข้อมูลก็ไม่ควรจะบันทึกลงไป อย่างเช่น หากไม่มีช่องให้กรอก “ชื่อ” และ “ที่อยู่” ก็ไม่ควรบันทึกข้อมูลเหล่านี้ลงในแผ่นบันทึกข้อมูล ไม่ควรมีการ “สร้าง” ข้อมูลใดๆขึ้นมาเองและบันทึกลงในแผ่นบันทึกข้อมูล

การเก็บรักษาข้อมูลที่เหมาะสมหมายถึงว่าได้มีการป้องกันด้านความปลอดภัยทั้งหลายในขณะที่เคลื่อนย้าย ขนส่งข้อมูลสู่สถานที่เก็บที่มีพื้นที่กว้างขวาง ผู้เก็บข้อมูลไม่ควรวางแผ่นบันทึกข้อมูลไว้ในที่ที่อาจเกิดการสูญหาย ถูกขโมย หรืออ่านโดยบุคคลภายนอก แผ่นบันทึกข้อมูลควรมอบให้กับบุคคลที่มีหน้าที่ในการเก็บรักษา และบุคคลนั้นย่อมต้องปฏิบัติตามคำแนะนำทั้งหมดเกี่ยวกับการปกป้องความลับของข้อมูล หากข้อมูลถูกเก็บในรูปแบบอิเล็กทรอนิกส์ก็ต้องปฏิบัติตามหลักการและกฎของความซื่อสัตย์ การปกป้อง การดูแลที่เหมือนกัน

ค. การเบี่ยงเบนออกจากวิธีปฏิบัติในการวิจัย

บางครั้งผู้เก็บข้อมูลไม่สามารถปฏิบัติตามวิธีปฏิบัติในการวิจัยอย่างเลี่ยงไม่ได้ และบางครั้งผู้เก็บข้อมูลก็ทำผิดพลาด สิ่งสำคัญคือต้องแจ้งให้หัวหน้าทีมวิจัยทราบเกี่ยวกับปัญหานั้น เพราะว่าหัวหน้าทีมวิจัยต้องรับผิดชอบในการรายงานปัญหาเหล่านั้นแก่คณะกรรมการพิจารณาการวิจัยประจำสถานบัน(IRB) ไม่ใช่เรื่องน่าอายในการรายงานปัญหา เพราะสิ่งเหล่านี้เกิดขึ้นได้ตลอดเวลา เป็นสิ่งจำเป็นที่ต้องรายงานปัญหาเหล่านั้น เพราะมันหมายความได้ว่าข้อมูลนั้นใช้การไม่ได้ หรืออาสาสมัครมีปัญหากับการวิจัย และยังหมายถึงว่าผู้ดูแลการวิจัยจะไม่สามารถเสร็จสิ้นการรายงานแก่ IRB ได้ด้วย

ผู้เก็บข้อมูลที่ดีจะแจ้งประเด็นเหล่านี้แก่ผู้ดูแลของพวกเขา และให้ผู้ดูแลตัดสินใจว่าจะมีวิธีดำเนินการอย่างไรต่อไป
